# 

**Published:** 1856-07

**Authors:** 


*A Work on Fractures.*—We notice in the proceedings of the American Medical Association, that Prof. Frank H. Hamilton, of Buffalo, obtained permission to publish his report in a separate form when completed.

This report, thus far, is, in certain respects, one of novel interest, and of very great value to the profession. Viewed simply as a work on the diagnosis and treatment of fractures, st possesses high merit, while in the very important matter of prognosis it is far superior to any other treatise. Telling the truth in a bold, yet candid way, it will commend itself to the good sense of prectical surgeons, however illy it may suit the pretensions of theorists. We are rejoiced to see such a work emanating from so competent and honest authority.

---

*Advertisement.*—We call the attention of our readers to the advertise ment of Mr. King, druggist, on the second page of the cover. Mr. King is a competent and honorable druggist, and has for many years had nothing to do with the patent-medicine trade; a fact which should commend him to the support of right-minded physicians.

---

*Consumption of Quinine.*—The Philadelphia Medical and Surgical Journal says that 300,000 ounces of quinine are annually consumed in the United States, meaning, it is presumed, imported, as there are two very large manufacturing establishments in this country which prepare it on an extensive scale, and which are not included in the computation of the Secretary of the Treasury, from which the above estimate is derived. It is worth, at the present time, from $3 to $4 the ounce.

---

*Lithotomy by a Crucial Incision.*—M. Caratheodory, surgeon to the Sultan, and Professor of Surgery at the Medical Schools of Constantinople, has lately operated in two cases by the bilateral section of Dupuytren, and in both instances found it necessary to resort to a second, vertical, incision through the centre of the prostate and the upper half of the sphincter ani, owing to the large size of the calculi. The patients were respectively twenty-six and thirty years of age, and both made good recoveries.

